# Surface Termination and Morphology of Single Crystal AlN by Ex Situ Chemical Treatment and In Situ MOCVD Process

**DOI:** 10.3390/mi17020242

**Published:** 2026-02-13

**Authors:** Yinghao Chen, Jun Zhang, Genhao Liang, Hongyi Yi, Lei Wang, Hao Ying, Lishan Zhao

**Affiliations:** 1 College of Advanced Interdisciplinary Studies, National University of Defense Technology, Changsha 410073, China; q904994398@163.com (Y.C.); zhangjun@nudt.edu.cn (J.Z.); 13055180205@163.com (H.Y.); wanglei09c@163.com (L.W.); 2 Hubei Jiufengshan Laboratory, Wuhan 430074, China; yinghao@jfslab.com.cn

**Keywords:** AlN, ex situ wet chemical cleaning, in situ hydrogen annealing, MOCVD, AFM, XPS

## Abstract

To achieve an atomically clean surface of single-crystal aluminum nitride (AlN) substrates, this study systematically evaluated the effects of each step in ex situ wet chemical cleaning (solvent, piranha solution, HF, HCl) and in situ hydrogen annealing. X-ray photoelectron spectroscopy (XPS) and atomic force microscopy (AFM) analyses revealed that while the combination of solvent and piranha solution exposed step morphology, its effectiveness in removing organic contaminants was limited. HF cleaning efficiently removed the oxide layer but introduced fluorine residues, whereas HCl cleaning left no chlorine residues but exhibited lower efficiency in oxide removal. In situ hydrogen annealing significantly reduced carbon and oxygen contamination, albeit accompanied by a transformation of the surface morphology from step to island mode. By modulating the low V/III ratio during low-temperature metal–organic chemical vapor deposition (MOCVD) growth, a controlled transition from 3D island growth to 2D step-flow growth was achieved. This research provides a basis for optimizing AlN substrate surface treatment, offering important insights for advancing nitride-based optoelectronic and power devices.

## 1. Introduction

Aluminum Nitride (AlN) possesses a wide bandgap of approximately 6.2 eV, a high breakdown field strength of up to 15.4 MV/cm, and an excellent thermal conductivity of 340 W/(m·K) at room temperature [1,2,3,4]. These outstanding properties make it a highly promising material for advanced technological applications, including high-power, high-frequency radio frequency (RF) and power electronic devices, deep ultraviolet (DUV) photonic devices, quantum computing and communication, and micro-electro-mechanical systems (MEMS) [5,6,7,8,9,10,11,12]. Free-standing single-crystal AlN (SC AlN) produced by physical vapor transport (PVT) exhibits relatively low defect densities [13,14]. It has demonstrated exciting performances in high-performance power electronic devices in recent years [11,12,15,16,17,18]. However, the industrial application of SC AlN still faces significant challenges. After the growth of SC AlN generated by PVT is completed, it needs to undergo processing such as cutting, grinding, and chemical–mechanical polishing (CMP). This process introduces impurity particles, damaged layers, and oxide layers due to mechanical stress and oxidants [19,20,21]. Additionally, the AlN surface spontaneously forms a native oxide layer when exposed to moist air [22,23,24]. These surface defects and impurities adversely affect the quality of subsequent thin film growth, leading to degraded device performance [24,25,26,27]. Therefore, the first challenge in utilizing SC AlN substrates is achieving an atomically clean surface.

Wafer surface cleaning is generally categorized into ex situ cleaning and in situ cleaning [23,28]. Ex situ cleaning typically includes wet cleaning and dry cleaning, with wet cleaning being widely adopted due to its straightforward process and cost-effectiveness. RCA cleaning has been extensively studied and optimized for other substrates, establishing a mature cleaning framework for these materials [29,30]. In contrast, research on wet cleaning for AlN substrates has predominantly focused on AlN thin films, with limited studies addressing the wet cleaning of SC AlN [24,31,32]. Although Lee et al. and Rich et al. have proposed wet cleaning procedures specifically for SC AlN, the underlying mechanisms and the impact of each step have not yet been thoroughly investigated [23,28]. In situ cleaning is performed within the deposition equipment. Metal–organic chemical vapor deposition (MOCVD) is widely used in industrial production due to its high growth rate. Rich et al. conducted an in situ high-temperature ammonia (NH_3_) annealing process on SC AlN in the MOCVD chamber, converting surface hydroxides into AlN [28]. It has been reported that high-temperature hydrogen (H_2_) annealing in the MOCVD environment helps improve substrate morphology [33,34,35]. However, there have been no reports on in situ high-temperature H_2_ annealing specifically applied to SC AlN.

SC AlN is regarded as a promising substrate for next-generation optoelectronic devices and electronic devices. Therefore, a clear understanding of the surface condition after treatment is essential for optimizing surface processing methodologies and can also provide valuable references for subsequent epitaxial growth optimization and device performance analysis. Although surface treatment strategies developed for AlN thin films may offer some insights, the distinct growth and processing histories of SC AlN grown by PVT could lead to different cleaning behaviors. This work builds upon the foundational studies by Lee and Rich, aiming to systematically reveal the impact of each ex situ cleaning step [23,28]. Furthermore, we demonstrate a practical cleaning procedure that incorporates in situ high-temperature H_2_ annealing in the MOCVD chamber. Additionally, the deposition behavior of AlN on AlN substrates is analyzed, achieving a transformation in substrate morphology. This method provides a viable strategy for the development of optoelectronic devices and electronic devices based on SC AlN.

## 2. Experiment

The SC AlN substrate used in this study was grown by PVT, from the 46th Research Institute of CETC [36]. The specifications were as follows: a diameter of 1 inch, a crystal orientation of (0001), and a dislocation density below 10^4^ cm^−2^. After chemical mechanical polishing (CMP), the surface roughness root mean square (RMS) was less than 0.3 nm.

**Figure 1:**
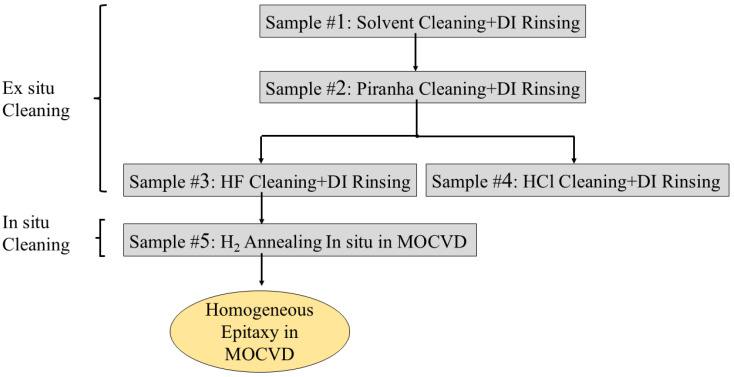
Flow chart of cleaning and epitaxy on SC AlN substrate in this work.

The whole experimental process in this work is shown in Figure 1. All ex situ chemical cleaning procedures were conducted under ultrasonic agitation at 70 °C. The samples underwent the respective ex situ chemical cleaning procedures:

1. Sample #1: 10 min acetone cleaning, 10 min isopropanol (IPA) cleaning, deionized (DI) water rinsing.
2. Sample #2: 10 min acetone cleaning, 10 min IPA cleaning, DI water rinsing, 10 min piranha solution cleaning (H_2_O_2_:H_2_SO_4_ = 1:3), DI water rinsing (three times).
3. Sample #3: 10 min acetone cleaning, 10 min IPA cleaning, DI water rinsing, 10 min piranha solution cleaning, DI water rinsing (three times), 3 min HF cleaning (2%), DI water rinsing.
4. Sample #4: 10 min acetone cleaning, 10 min IPA cleaning, DI water rinsing, 10 min piranha solution cleaning, DI water rinsing (three times), 3 min HCl cleaning (5%), DI water rinsing.

Upon completion of the ex situ chemical cleaning process for each sample, it was immediately dried using a nitrogen (N_2_) stream. This was followed by vacuum packaging to prevent recontamination. This two-step procedure ensured the sample’s integrity until the package was unsealed within the glovebox integrated with the characterization or growth equipment.

After the ex situ cleaning process of Sample #3, it was transferred to the MOCVD (Satur N800, NAURA Technology Group Co., Ltd., Beijing, China) for in situ cleaning. The operations performed on the sample are as follows:

Sample #5: 10 min acetone cleaning, 10 min IPA cleaning, DI water rinsing, 10 min piranha solution cleaning, DI water rinsing (three times), 3 min HF cleaning (2%), DI water rinsing, N_2_ drying, vacuum packaging, unsealing in MOCVD glovebox, H_2_ annealing in situ treatment.

Sample #5 was treated with H_2_ at 1050 °C and 60 Torr for 10 min, and the H_2_ flow was 150 slm (standard liters per minute).

Following the completion of Sample #5, AlN homoepitaxy was performed. Trimethylaluminum (TMAl) and NH_3_ were used as precursors for Al and N atoms, with H_2_ as the carrier gas. The AlN layer was grown at a low temperature with the V/III ratio (the molar flow ratio of NH_3_ to TMAl precursors). The specific growth parameters are temperature of 1100 °C, pressure of 50 torr, V/III ratio of 75, and thickness of approximately 100 nm.

The surface chemical composition and chemical bonding states of the AlN substrate were characterized by X-ray Photoelectron Spectroscopy (XPS, Thermo Fisher Scientific/NEXSA, Waltham, MA, USA). XPS analysis was performed using a system equipped with a monochromatic Al *Kα* X-ray source (*hν* = 1486.6 eV) operated at 15 kV. The base pressure in the ultra-high vacuum (UHV) analysis chamber was maintained below 1 × 10^−10^ Torr. The energy scale of the spectrometer was calibrated using the Au 4*f*_7_/_2_ peak at a binding energy of 84.0 eV from a standard sample. All reported binding energies were referenced to the adventitious carbon (C–C/C–H) C 1s peak set at 284.8 eV to correct for charging effects. Before peak fitting, a smart background subtraction was applied to all high-resolution spectra. Following background removal, the spectra were deconvoluted into their individual chemical components using a product of Gaussian-Lorentzian functions. To ensure physical significance, the full width at half-maximum (FWHM) for peaks belonging to the same chemical species was constrained within a reasonable range between 1.0 and 2.0 eV. The synthetic curve was iteratively optimized by adjusting the peak positions, areas, and FWHM to achieve the best possible fit to the experimental data. The surface morphology of the AlN substrate was characterized using atomic force microscopy (AFM, Dimension Icon, Bruker Co., Ltd., Billerica, MA, USA) performed in tapping mode. The obtained AFM images were processed with second-order flattening to eliminate background noise caused by sample tilt and were subsequently smoothed using a Gaussian filter. The crystal quality of the AlN substrate and epitaxial layer was characterized by high-resolution X-ray diffraction (HR-XRD, Rigaku SmartLab3KW, Rigaku, Tokyo, Japan). Rocking curve (ω-scan) measurements were performed on the symmetric (002) and asymmetric (102) reflections. The obtained curves were normalized, smoothed using the Akima method, and their FWHM were determined by fitting with a Gaussian function.

Chemicals used were: acetone (AR grade—Tianjin Fuyu Fine Chemical Co., Ltd., Tianjin, China), IPA (AR grade—Sinopharm Chemical Reagent Co., Ltd., Shanghai, China), 98% H_2_SO_4_ (AR grade—Chengdu Kelong Chemical Co., Ltd., Chengdu, China), 30% H_2_O_2_ (AR grade—Sinopharm Chemical Reagent Co. Ltd.), 36% HCl (AR grade—Chengdu Kelong Chemical Co., Ltd.), and 2% HF (AR grade—Chengdu Kelong Chemical Co., Ltd.).

## 3. Results and Discussion

**Figure 2:**
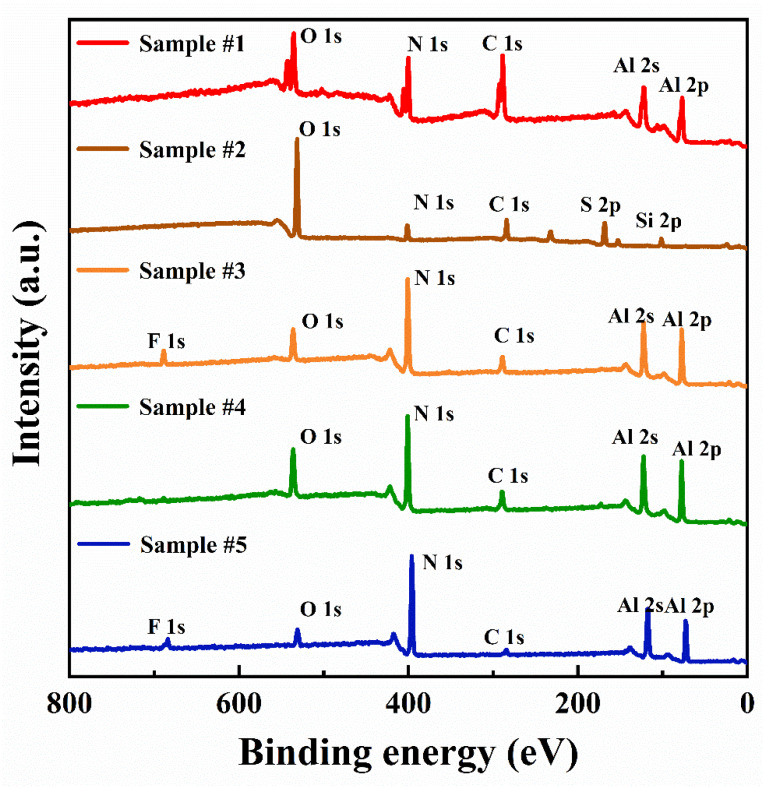
XPS survey spectra of SC AlN substrates under different processing steps.

XPS survey scans were performed on all samples subjected to both ex situ and in situ cleaning processes, with the resulting spectra shown in Figure 2. As expected for AlN, all spectra exhibited characteristic peaks intrinsic to the material, including Al 2s, Al 2p, and N 1s. Peaks associated with native contamination (C 1s, O 1s), residues introduced during cleaning (S 2p, F 1s), and an impurity inherent to the AlN material (Si 2p) were also identified. However, the relative atomic concentrations of the detected elements varied markedly across samples subjected to different cleaning steps, as quantified in Table 1.

**Figure 3:**
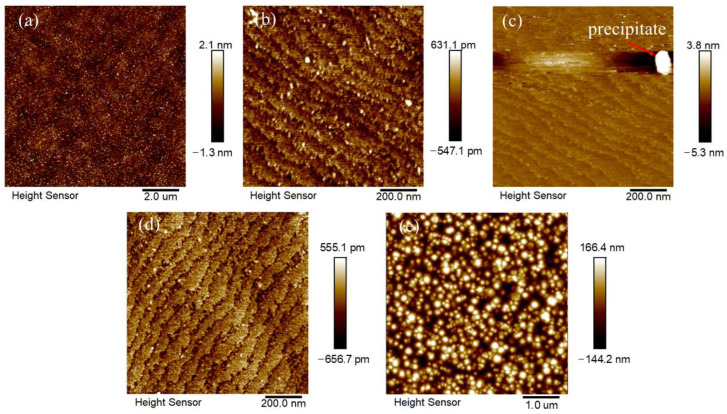
The surface morphology of each sample: (**a**) Sample #1, (**b**) Sample #2, (**c**) Sample #3, (**d**) Sample #4 and (**e**) Sample #5. Notably, a prominent white speck was observed in the upper-right region of image (**c**). It was significantly larger than the surrounding topographic features and reproduced with probability in repeated measurements.

Acetone and IPA are commonplace solvents for removing organic residues [23,28,29]. Although acetone dissolves contaminants and IPA rinses and dries quickly, this method failed to produce a clean AlN surface. Figure 3a shows significant residual contamination, consistent with the high carbon concentration measured for Sample #1 in Table 1. The unsatisfactory effect of this method is explained by the high polarity and surface energy of AlN, which cause strong binding of organic molecules, preventing their effective removal by solvent washing [38,39].

As shown in Figure 3b, Sample #2 after piranha solution cleaning exhibited a distinct step morphology alongside significant contaminant residues. This observation is consistent with previous reports, revealing the dual effects of piranha solution cleaning on AlN substrate surface treatment [23,31]. Due to the similar crystal structure and characteristics of sapphire and AlN, they may have high referenceability in terms of step formation mechanism. The mixture of phosphoric acid and sulfuric acid, due to its specific anisotropic etching characteristics, more readily induces step structures on AlN substrate surfaces [40,41]. In contrast, the piranha solution used in this study, while effective in decomposing organic contaminants through strong oxidation, requires precise control of key parameters such as solution temperature and processing duration to achieve ideal step formation. It is noteworthy that the step structure observed after piranha cleaning may be related to secondary effects of selective etching on specific AlN crystal planes [40,42]. The quantitative analysis in Table 1 reveals the limitations of the piranha cleaning step in terms of contaminant removal. The high residual carbon content (22.20 at.%) indicates ineffective organic removal. This is likely a direct consequence of the previous solvent cleaning step being unsatisfactory, which left behind a heavily contaminated surface. The piranha solution was unable to completely oxidize this organic layer. Concurrently, the cleaning introduced significant new contamination in the form of sulfur residues (12.10 at.%) and oxygen residues (47.12 at.%), suggesting that the solution’s strong oxidizers promoted the adsorption of sulfate species onto the substrate [40,41]. Thus, the step not only failed to fully clean the surface but also added an amorphous cover layer or “dirt”. Particularly noteworthy is the severe deviation from the ideal stoichiometric ratio observed after piranha treatment. This anomaly indicates that the cleaning process may have caused significant alterations in surface composition.

Studies have shown that HF and HCl solutions can effectively remove the native oxide layer from AlN surfaces [23,31,32]. As shown in Figure 3c,d, the samples after HF and HCl cleaning (Sample #3 and Sample #4) both maintained clear atomic step morphology. HF and HCl treatment effectively removed the oxide layer without significantly damaging the substrate’s crystal structure. The Al/N ratio of Sample #3 and Sample #4 in Table 1 is slightly less than 1, confirming the effectiveness of the HF and HCl cleaning step in removing the oxide layer on the aluminum-rich surface. Regarding oxide removal efficiency, the oxygen content on Sample #3 was reduced to 11.10 at.%, significantly lower than the 16.49 at.% on Sample #4, demonstrating that HF exhibited superior efficiency in removing the oxide layer on the AlN surface. At the same time, HF and HCl cleaning successfully eliminated the sulfur contamination introduced by the previous piranha solution step and further reduced the carbon content. This result indicates that HF and HCl treatment can effectively remove pollutants remaining from the previous process.

However, the two acid treatments showed significant differences in residue control. XPS analysis confirmed the presence of 3.05 at.% fluorine residue on the Sample #3 surface, consistent with literature reports that HF cleaning tends to introduce fluorine contamination [31,32]. The reaction between HF and both the bulk AlN and surface oxides is considered as follows [43]:

Al_2_O_3_(s) + 6HF(aq) = 2AlF_3_(s) + 3H_2_O(aq) (1)

AlN(s)+ 4HF(aq) = AlF_3_(s) + NH4^+^(aq) + F^−^(aq) (2)

The insoluble aluminum fluoride (AlF_3_) formed from the reaction creates a passivation layer on the surface, which inhibits further oxidation of AlN. However, in regions where AlF_3_ becomes locally supersaturated and accumulates, while the rest of the surface maintains intact atomic steps, it leads to the formation of distinct particulate residues, as shown in Figure 3c. The reaction between HCl and surface oxides is considered as follows [44]:

Al_2_O_3_(s) + 6HCl(aq) = 2AlCl_3_(aq) + 3H_2_O(aq) (3)

The reaction products are water-soluble salts that can be completely removed by rinsing, resulting in no chlorine residue on Sample #4. The trace sulfur residue found in Sample #4 indicates that the available Cl^−^ ions were preferentially consumed in removing sulfate groups introduced during the previous cleaning step. It can be inferred that their limited reactivity was exhausted in this process, which not only left some sulfur impurities behind but also prevented the incorporation of chlorine residues.

As shown in Figure 3e, after in situ hydrogen annealing at 1050 °C in the MOCVD chamber, the surface morphology of Sample #5 transitioned from a step structure to an island pattern, accompanied by a significant reduction in carbon and oxygen content (C: 6.09 at.%, O: 5.99 at.%). This morphological transformation primarily stems from the thermodynamically driven equilibrium reactions in the hydrogen environment [28,34]:

AlN(s) + 3H* ↔ Al(l) + NH_3_ (4)

Al_2_O_3_(s) + 6H* ↔ 2Al(l) + 3H_2_O(g) (5)

Under the high temperature environment of the MOCVD chamber, H_2_ undergoes thermal dissociation to generate atomic hydrogen (H*), which serves as the core active species in the etching process and dominates the chemical reactions on the AlN surface [45]. Endowed with high reactivity, H* cleaves Al-N bonds and Al-O bonds on the surface. On one hand, it combines with N atoms and O atoms to form NH_3_ and H_2_O. On the other hand, the released Al atoms form liquid Al since the temperature exceeds its melting point (660 °C). Liquid Al tends to accumulate in surface defects (e.g., holes) to create an Al-rich and H-rich local environment. The increased Al/N ratio of 1.06 for Sample #5 in Table 1 confirms an aluminum-enriched surface state. As theoretically confirmed by Akiyama et al. and experimentally observed by Pristovsek et al., this environment promotes the formation of a metallic Al bilayer on the (0001) AlN surface [46,47]. Similar to the N transport mechanism in InGaN under a metal bilayer [48], the Al bilayer acts as a transport medium to facilitate the migration of N atoms from the bulk AlN to the surface, further catalyzing AlN decomposition. The (0001) AlN surface exhibits intrinsic “slow growth but fast etching” characteristics. Under near equilibrium H* etching conditions, it destroys the original ordered step structure and pushes the surface to an island structure. Notably, 2.49 at.% fluorine residue persists, possibly shielded from complete desorption by the overlying aluminum-rich regions [31].

**Figure 4:**
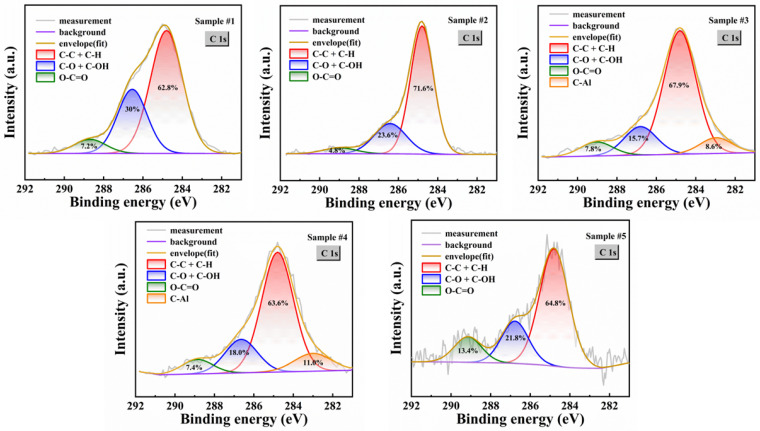
C 1s XPS spectra of SC AlN substrates under different processing steps. The contents of different binding structures are noted within the sub-peaks.

To further elucidate the specific effects of each cleaning step on the surface chemistry, we performed detailed peak fitting on the high-resolution XPS spectra of C 1s, O 1s, N 1s, Al 2p, and F 1s. As shown in Figure 4, the adventitious carbon was deconvoluted into three typical carbon species: aliphatic carbon (C–C/C–H) at a binding energy of 284.8 eV, single-bond oxygen-containing functional groups (C–O/C–OH) at 286.3 eV, and carboxyl groups (O–C=O) at 288.8 eV [49,50]. Notably, a distinct C–Al characteristic peak near 282.8 eV is observed in Sample #3 and Sample #4 [51]. The analysis results indicate that aliphatic carbon is the dominant component of carbon contamination in all samples. The reason for this phenomenon is that the (0001) AlN substrate used in this article has a large number of unsaturated Al dangling bonds on the surface. Al atoms exhibit strong chemical bonding affinity with C atoms, and these dangling bonds act as active sites to chemically adsorb organic molecules (e.g., aliphatic carbon) from the environment, leading to sustained carbon pollution [52]. Due to the high polarity of the AlN surface, Sample #1 exhibits a relatively high proportion of oxygen-containing polar organics (C–O and O–C=O). In Sample #2, the proportion of oxygen-containing organics decreases, indicating that the piranha solution failed to fully exert its oxidative properties, which may be a reason for its unsatisfactory cleaning effectiveness against organic contaminants. The emergence of the C–Al characteristic peak in Sample #3 and Sample #4 suggests that HF or HCl treatment exposes aluminum atoms from the bulk AlN and thus effectively removes the surface oxide layer. These exposed aluminum atoms react with residual low-binding-energy organic carbon (C–C and C–O) on the surface, forming C–Al bonds. Although the total carbon content is significantly reduced in Sample #5 after high-temperature hydrogen annealing, its carbon species distribution resembles that of Sample #1. The result confirms that it enables the surface carbon species to return to a cleaner intrinsic state by deeply removing various organic pollutants.

**Figure 5:**
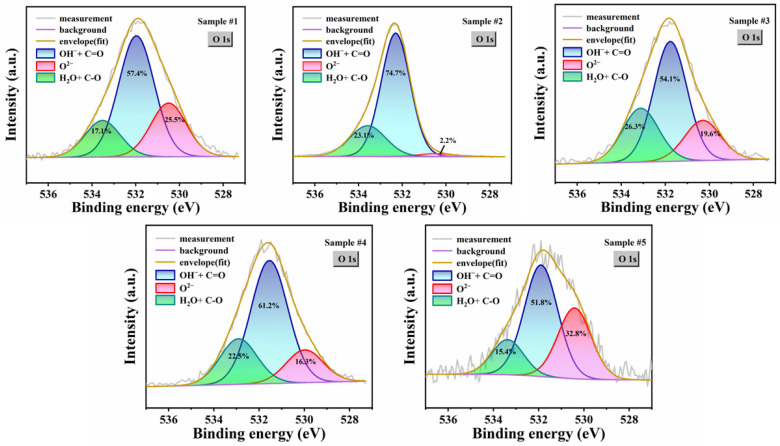
O 1s XPS spectra of SC AlN substrates under different processing steps. The contents of different binding structures are noted within the sub-peaks.

Research indicates that the surface oxides of SC AlN substrates primarily exist as Al_2_O_3_, Al(OH)_3_, and AlOOH, and the surface is prone to physically adsorbing water or intercalated water [20,28]. Considering the presence of organic pollutants with oxygen-containing functional groups on the surface of AlN. As shown in Figure 5, the O 1s spectra can be deconvoluted into three components: OH^−^ and C=O species at 531.7 eV, O^2−^ species at approximately 530.5 eV, H_2_O and C–O species at 533.2 eV [21,28]. Since all ex situ cleaning processes involve polar DI water treatments, complete removal of surface-adsorbed water remains challenging even after nitrogen blowing. Therefore, all samples exhibit the H_2_O characteristic peak. Under such moist conditions, oxides predominantly exist in Al(OH)_3_ and AlOOH forms. Due to oxidation of organic matter, sample #2 exhibits the highest C–C and C=O signals. The extremely low proportion of O^2−^ in Sample #2 is mainly due to the strong oxidative effect of the piranha solution, which induces hydroxylation of the AlN surface to form a hydrophilic surface [53]. The O^2−^ is largely converted into OH^−^, and the hydrophilic surface further adsorbs more water, resulting in a significant increase in H_2_O content compared to Sample #1. The higher proportion of O^2−^ in Sample #3 than in Sample #4 can be explained by the fluoride-induced passivation layer formed during HF cleaning, which inhibits the conversion of Al_2_O_3_ to Al(OH)_3_ in moist environments [31]. In Sample #5, the increased proportion of O^2−^ indicates that the high-temperature environment facilitates the stabilization of Al_2_O_3_. However, even after the high-temperature treatment, the characteristic peak of H_2_O cannot be eliminated [24]. On the one hand, the H_2_ gas source inevitably contains trace amounts of water. On the other hand, the reaction between surface oxides and H_2_ during annealing generates H_2_O, as represented by Equation (5).

**Figure 6:**
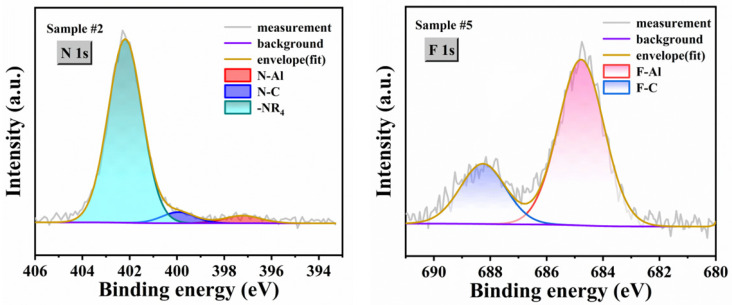
N 1s XPS spectrum of Sample #2 and F 1s XPS spectrum of Sample #5. -NR_4_ in the figure represents a nitrogen-containing charged group.

To clarify the previously ambiguous surface chemical states, we performed supplementary high-resolution peak fitting on the N 1s spectrum of Sample #2 and the F 1s spectrum of Sample #5, as shown in Figure 6. The N 1s spectrum of Sample #2 not only exhibited the intrinsic N–Al bonds from SC AlN substrate and possible N–C bonds introduced by CMP, but also showed characteristic signals corresponding to protonated nitrogen species (–NR_4_) at higher binding energies [20,54,55]. These species likely originate from the protonation of N atoms on the AlN surface and nitrogen-containing groups in contaminants under the acidic environment of the piranha solution [56]. It is noteworthy that these protonated nitrogen species account for the majority of the total spectral area, reasonably explaining the stoichiometric imbalance observed in Sample #2, where the N content significantly exceeds the Al content. In the analysis of Sample #5, the coexistence of F–Al and F–C characteristic peaks was detected [31,57]. The formation of F–C bonds can be attributed to carbon contamination introduced by the graphite susceptor in the MOCVD system during high-temperature processing, which reacts with the AlN surface under annealing conditions to form F–C bonds. This side reaction may have partially inhibited the complete removal of fluorine elements under high-temperature conditions.

**Figure 7:**
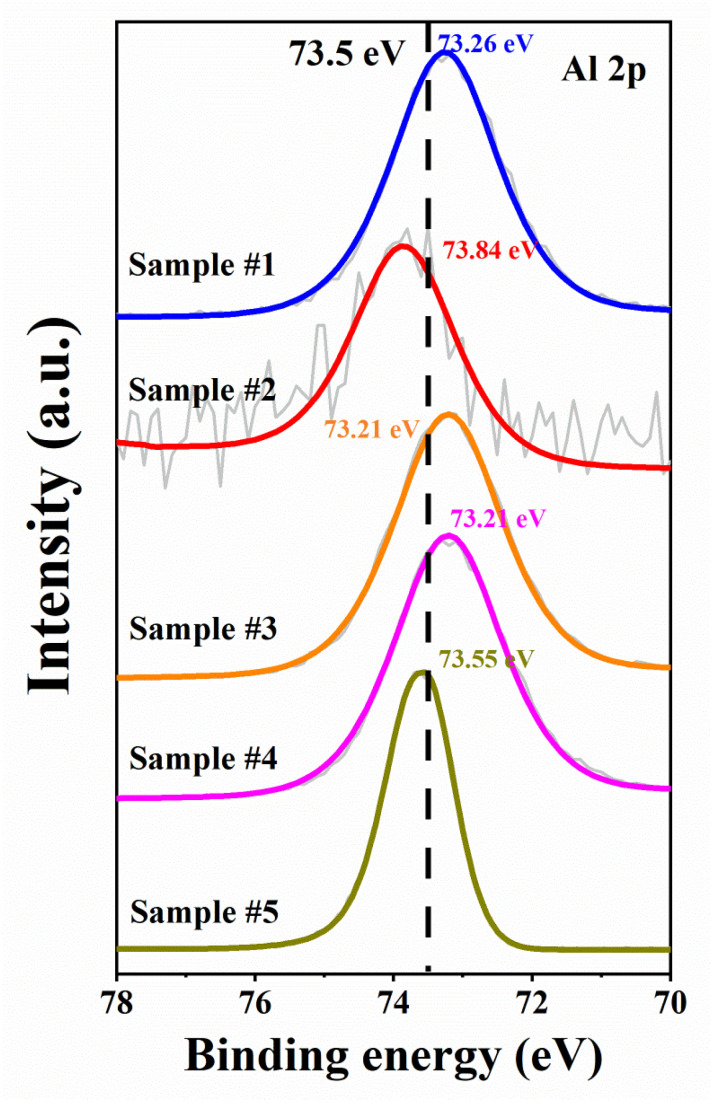
Al 2p XPS spectra of SC AlN substrates under different processing steps. The dash line of 73.5 eV is the binding energy position of Al-N bond [20,21,59]. The gray line are measurement.

Building on the understanding of each sample’s surface state from previous analyses, we further analyzed the Al 2p spectra, as shown in Figure 7. Here, we employed binding energy shift analysis to interpret changes in the surface chemical environment. The variation in Al 2p peak position is a comprehensive result of multiple factors, including the relative content of surface oxidation states, carbon-related bonding interactions, and subtle changes in surface charge environment, rather than being solely attributed to a single factor. Sample #1 shows a slight shift toward lower binding energy, which is associated with the effect of residual organic contaminants and surface oxides. Sample #2 exhibits the highest binding energy among all samples, consistent with its severe surface oxidation and the formation of protonated species that induce a positively charged surface environment. Sample #3 and Sample #4 both show peak positions shifted toward lower binding energy relative to Sample #2, which aligns with the effective reduction in surface oxides and sulfur residues by acid treatment. Though residual organic carbon still exerts a weak influence on the local electron environment of Al atoms. Sample #5 is closest to the intrinsic Al–N bond position of bulk AlN. This is attributed to the significant reduction of carbon (6.09 at.%) and oxygen (5.99 at.%) contamination, minimizing the interference of external factors on the Al chemical environment. It is worth noting that no effect of the Al-Al bond was detected in Sample #5, which should shift the peak position towards lower binding energy. This may be due to the weakening of AlN cracking during the cooling process of liquid Al, and the continuous flow of H_2_ causing the evaporation of liquid Al [58]. Ultimately, there is no residual liquid aluminum on the surface, or the content is very low.

The Sample #5 exhibits an island morphology after high-temperature H_2_ annealing. Although it can provide nucleation sites for subsequent functional layer growth, dislocations are prone to form during island coalescence, and these dislocations will propagate upward along the epitaxial direction, severely degrading device performance. In contrast, maintaining a step morphology and achieving a 2D growth mode can theoretically significantly suppress dislocation generation. Traditionally, 2D growth of AlN epitaxial layers typically relies on high temperatures above 1200 °C [60]. This study proposes a low-temperature MOCVD growth strategy to realize the transformation from island to step morphology on SC AlN surfaces by regulating the V/III ratio.

**Figure 8:**
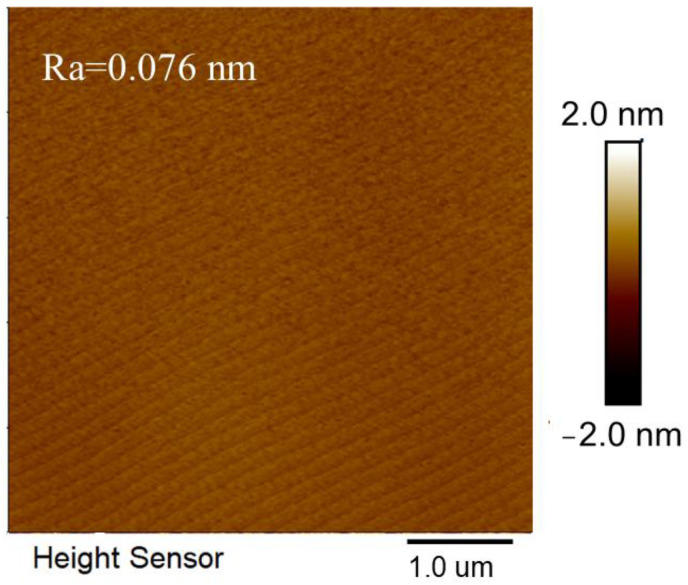
AFM images of AlN epitaxial layers deposited.

**Figure 9:**
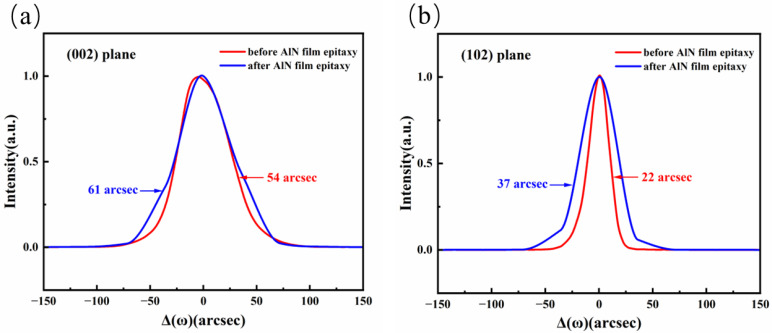
HR-XRD rocking curves of (**a**) (002) and (**b**) (102) planes before and after AlN film epitaxy.

By precisely adjusting the V/III ratio, the morphology transformation can be achieved at a relatively thin epitaxial thickness (~100 nm). As shown in Figure 8, the surface is completely transformed into a regular step morphology with a roughness of 0.076 nm, a step width of ~200 nm, and a step height of ~0.5 nm, which matches the slight miscut angle of the AlN substrate. The HR-XRD rocking curves of the AlN film before and after epitaxial growth are shown in Figure 9. The FWHM values of the (002) and (102) planes for the epitaxial AlN layer are 61 arcsec and 37 arcsec, respectively. Compared with the pristine SC AlN substrate (54 arcsec for (002) and 22 arcsec for (102) before epitaxy), the epitaxial layer maintains the high crystalline quality of the AlN substrate. Although surface defects or slight fluctuations in the growth process may result in minor defects during regrowth, SC AlN still exhibits excellent crystal quality under the cleaning scheme and growth conditions used in this study.

Existing theories indicate that MOCVD of AlN epitaxial layers is a non-equilibrium process driven by aluminum vapor supersaturation. Although this theory was proposed based on ideal planes, this experiment confirms that it is also applicable to island substrates. As the growth driving force, vapor supersaturation ($\sigma_{Al}$) is defined as [28,61]:

(6) $$\sigma_{Al}=\frac{P_{Al}^{0}\;-{\;P}_{Al}}{P_{Al}}\;\;$$

where $P_{Al}^{0}$ is the input partial pressure of the aluminum source, and $P_{Al}$ is the equilibrium vapor pressure of aluminum. At low temperatures, $\sigma_{Al}\;$ should be at a relatively high level, which tends to induce island growth. However, this study compensates for the effect of high $\sigma_{Al}\;$ by reducing the V/III ratio. Under a low V/III ratio, the supply of N atoms was reduced, which inhibits the excessive aggregation of Al atoms. It promotes Al atom migration along the substrate surface and filling of inter-island gaps, and ultimately achieves step growth.

## 4. Conclusions

The ex situ cleaning process began with standard organic solvent cleaning (acetone and IPA), which leaves significant carbon contamination due to the high surface energy of AlN. Subsequent treatment with a piranha solution successfully exposed a distinct step morphology. Due to the large amount of organic residue in the previous step, the effect was not significant in the oxidation treatment of organic matter. At the same time, a large amount of new pollution has been introduced, including sulfur residues and highly oxidized surfaces, accompanied by protonation of nitrogen atoms. The subsequent acid treatments demonstrated a critical trade-off. HF cleaning efficiently removed the native oxide layer but left behind a fluorine residue in the form of an insoluble AlF_3_ passivation layer. In contrast, HCl cleaning left no chlorine residues but was less efficient in oxide removal. The surface morphology after these acid steps remained favorable, preserving the atomic step structure.

The process was significantly advanced by introducing an in situ high-temperature hydrogen annealing step within the MOCVD chamber. This treatment dramatically reduced both carbon and oxygen contamination to 6.09 at.% and 5.99 at.%, respectively. However, this improvement in chemical purity came at a morphological cost, as the thermodynamically driven reaction with hydrogen transformed the surface from a step structure to an island pattern, accompanied by an aluminum-enriched surface. To address this morphological shift, the study modulated the growth conditions during subsequent low-temperature AlN homoepitaxy. By controlling the low V/III ratio, a controlled transition from 3D island growth to 2D step-flow growth was achieved. The low V/III ratio promotes adatom migration and gap filling between islands, resulting in a regular step morphology with a remarkably low roughness of 0.076 nm. In conclusion, this work provides a holistic and optimized strategy for AlN substrate preparation. It clarifies the mechanisms and trade-offs of each cleaning step, demonstrating the powerful yet complex effects of in situ hydrogen annealing. Then, a low V/III ratio was used to restore the ideal morphology during MOCVD growth. This comprehensive approach offers vital insights for the development of high-performance devices on SC AlN substrates.

## Figures and Tables

**Table 1:** Atomic ratio of elements of SC AlN substrates under different cleaning steps. The table lists only the relative concentration of Si. Other impurities, such as B and P, are omitted due to their negligible levels [1,37].

Sample	Al (at.%)	N (at.%)	C (at.%)	O (at.%)	S (at.%)	F (at.%)	Si (at.%)
Sample #1	20.04	18.31	38.75	19.33	0	0	3.56
Sample #2	1.49	11.27	22.20	47.12	12.10	0	5.83
Sample #3	31.91	36.33	14.08	11.1	0	3.05	3.53
Sample #4	31.9	33.99	14.47	16.49	0.25	0	2.89
Sample #5	43.57	41.19	6.09	5.99	0	2.49	0.67

## Data Availability

The data presented in this study is available upon request from the corresponding authors.
